# Searching and generating test inputs for mutation testing

**DOI:** 10.1186/2193-1801-2-121

**Published:** 2013-03-21

**Authors:** Mike Papadakis, Nicos Malevris

**Affiliations:** 1 Interdisciplinary Center for Security, Reliability and Trust, University of Luxembourg, Luxembourg, Luxembourg; 2 Department of Informatics, Athens University of Economics and Business, Athens, Greece

**Keywords:** Test case generation, Search based testing, Mutation testing

## Abstract

Mutation testing is usually regarded as an important method towards fault revealing. Despite this advantage, it has proved to be impractical for industrial use because of its expenses. To this extend, automated techniques are needed in order to apply and reduce the method’s demands. Whilst there is much evidence that automated test data generation techniques can effectively automate the testing process, there has been little work on applying them in the context of mutation testing. In this paper, search-based testing is used in order to effectively generate test inputs capable of revealing mutants. To this end, a dynamic execution scheme capable of introducing and guiding the search towards the sought mutants is proposed. Experimentation with the proposed approach reveals its superiority from the previously proposed methods. Additionally, the framework’s feasibility and practicality of producing mutation based test cases are also demonstrated.

## Introduction

Software testing can account for more than half of the cost of the software under development. As the main purpose is to reduce such an excessive cost, the testing activity should incorporate effective and efficient methods experiencing the highest possible level of automation. The test data generation process plays a crucial role in both the effectiveness and efficiency of the software testing phase. Unfortunately, as it is evident from the current practice, the level of automation achieved to date is not as high as it ought to be, thus resulting in a rather low quality testing activity due to the unavoidably high cost of the imperative laborious manual activity. Hence, the need for producing the required test data automatically is essential in order to increase the test thoroughness and to reduce the testing expenses at the same time.

Testing quality is usually measured by the test adequacy criteria. Adequacy criteria, often referred to as coverage criteria, pose certain requirements that should be fulfilled by the test cases. Mutation testing or mutation analysis, is a fault-based technique introduced by Hamlet (1977) and DeMillo et al. (1978). Mutation analysis makes alterations, called mutants, to the code under test based on a set of simple syntactic rules called mutant operators. The purpose of injecting mutants into programs is to both guide the generation of test cases to reveal them on the one hand and to assess the test data quality on the other. To this extent, testing seeks to reveal the mutants, which when detected are termed “killed” and “live” in the opposite case. Testing adequacy is measured using the mutation score, defined as the ratio of the number of the killed mutants to the entire number of candidate mutants reduced by the number of equivalent ones. Equivalent mutants are those mutants that cannot be killed by any test case. This is an analogous form of the infeasibility element problem encountered in structural testing (Offutt and Pan 1997).

The strength of the method relies on the hypothesis - ability of the introduced mutants to produce realistic faults. In a study made by Andrews et al. (2006), this hypothesis is reinforced. Additionally, mutation has been empirically found to be more effective than other structural testing criteria (Offutt and Untch 2001) and it provides significant assistance in various debugging activities (Papadakis and Le Traon 2012). Thus, it is evident that developers can benefit from applying mutation testing. Although powerful, mutation lacks practical use. The practical use of an adequacy criterion requires the automated generation of test cases according to its requirements. This can prove to be a very laborious task (Offutt and Untch 2001) while, little has been done into automating effectively the mutation-based test production. Additionally, little has also been done into automating effectively the production of the sought mutation based test cases. This constitutes the main issue of the present research.

Automating mutation testing requires the production of the candidate mutant programs and their execution with the candidate test cases. This can be efficiently automated with the mutant schemata approach (Untch et al. 1993), (Papadakis et al. 2010), (Ma et al. 2005) which embeds all the candidate mutants into one schematic meta-program and thus, all tests are executed against this schematic program. Test execution poses an additional barrier to mutation analysis as it requires test cases to be executed against all live mutants. To effectively reduce the execution time required for mutation, alternative methods called weak or firm mutation (Jia and Harman 2010), (Howden 1982), (Offutt and Untch 2001) have been proposed. According to these methods the program execution may stop after the mutated or a succeeding program expression. Evaluation of the mutant can be performed by checking the program state at the stopping execution statement. Thus, remarkable execution savings can be achieved. Additionally, by utilizing weak mutation and mutant schemata techniques all the weakly killable mutants can be recorded with only one execution run (Papadakis et al. 2010), (Papadakis and Malevris 2011). The framework proposed in this paper takes advantage of this fact and executes only the weakly killed mutants in order to determine the strongly killed ones. Thus, the mutant execution cost is kept to a low level.

The practical use of an adequacy criterion requires the automated generation of test cases according to its requirements. This can be a very laborious task (Offutt and Untch 2001) for any selected criterion including mutation. Recently, search based optimization techniques and tools have succeeded in automating the test case generation activity effectively i.e. (Harman and McMinn 2007) and (Lakhotia et al. 2010). This paper introduces an automated framework that produces test cases based on strong mutation testing. In the proposed framework, the mutants are automatically generated based on a novel version of the mutant schemata technique (Untch et al. 1993) for performing both mutation and search based testing. The use of mutant schemata for mutation test data generation purposes has also been investigated by (Papadakis and Malevris 2011), (Papadakis et al. 2010) in the context of weak mutation, utilizing existing structural testing tools, and in the context of strong mutation using dynamic symbolic execution (Papadakis and Malevris 2010a). Here the proposed approach incorporates a hill climbing algorithm known as the alternating variable method (AVM) proposed by Korel (1990) for searching and producing the sought test cases for strong mutation. The choice of the AVM method was due to its simplicity and the high expected effectiveness in the context of structural testing (Harman and McMinn 2007) and (Lakhotia et al. 2010).

The origins of the present approach are due to the utilized dynamic fitness scheme. Thus, it becomes possible to effectively direct the search process towards reaching, infecting and impacting the targeted mutants. A performed case study suggests that it can be more effective than random testing and a previously proposed approach (Ayari et al. 2007).

The contribution of the present work can be summarized into the following points:

-  A novel scheme able to perform both mutation and search based testing.
-  A novel fitness function for strongly killing mutants.
-  A novel dynamically adjusted fitness scheme able to improve the effectiveness of search based approaches.

The rest of this paper is organized as follows: Section 2 presents the proposed system by detailing the proposed approach. In Section 3 presents and analyzes the obtained results from the application of the proposed approach. Sections 4 and 5 discuss the relevance and the benefits of the application of the proposed system with previously proposed systems and approaches. Finally in section 6 conclusions and future directions are given.

## Framework description

The proposed framework tries to effectively automate the production and evaluation of mutation based test data. The framework embeds all the candidate mutants into one schematic meta-program suitable for both executing mutants and recording the required test cases fitness calculations. Then it produces test cases according to the alternating variable method (Korel 1990) guided by the schematic program. In the succeeding subsections details of the framework are given.

### Generating mutants

Dynamic approaches are based on the information gained through dynamic program runtime execution. In the context of structural testing the programs under test host all the needed information in their structure and thus, it is straightforward to implement a monitoring mechanism for the data evolution purpose. In the context of mutation, there is a special need for unifying both the original’s and the mutants’ runtime information. The difficulties originate from the mutations’ method nature, as the needed information is spanned across the original and the various mutants versions (Papadakis et al. 2010), (Papadakis and Malevris 2009) programs. To efficiently overcome this difficulty a special form of the mutant schemata technique was employed in order to unify all the mutation analysis requirements into a unique version suitable for the test evolution representation. This approach was initially introduced in (Papadakis et al. 2010) in the context of using existing test data generation tools for performing mutation. Here the technique has been extended in order to effectively guide the test data generation process. This is achieved by embedding a fitness guide and evaluation inside the schematic functions.

The Mutant Schemata Generation (MSG) (Untch et al. 1993) technique encodes into one meta-program all the introduced mutants. This is achieved by appropriately replacing each pair of operands participating in an operation with a call to a schematic function, with this pair of operands as parameters (e.g. a > b becomes RelationalGT (a, b)). Expanding the suggestions of the MSG approach, the evaluation of the mutants’ execution and all the required fitness calculations are performed within the schematic function. This as it is shown in (Papadakis et al. 2010) reflects the killing mutants problem to a path - branch coverage problem. By incorporating the mutant evaluation into the schematic function, the necessary conditions for killing each considered mutant are also embedded. These conditions, which take the following expression, are formed as decisions in the schematic function. These conditions are of the following form:1

The above decision expression (1) consists of two possible outcomes (i.e. the original is either equal to the mutated or not). Thus, entailing the introduction of true (mutant is killed) and false (mutant is alive) cases. Measuring the closeness of making the above decision (1) true, results in an effective measure of the test case fitness according to the weak mutation testing criterion (Papadakis et al. 2010) and forms a part of the proposed fitness function (section 2.4). After the mutants’ evaluation point, the program execution continues in order to evaluate the mutant’s output and its propagation fitness, as it is required by strong mutation.

### Executing mutants with tests

The present approach takes advantage of the unified representation of all mutants and their killing conditions into one meta-program. Based on the use of the introduced schemata technique, mutant execution can be performed straightforwardly utilizing only one program. This is a direct consequence of the parameterized introduced mutants (Untch et al. 1993), (Papadakis and Malevris 2011). Additionally, the fitness function calculations have also been embedded into the schematic classes. Thus, test execution requires only an initialization of the mutant schemata class at the beginning of the execution and an additional call to the calculation function at the end in order to produce all the required fitness calculations.

The proposed system employs the reflection mechanism of the Java language in order to execute the meta-program with the produced test cases and extracts the fitness function calculations. As the proposed system performs a different search per live mutant, it executes all the live mutants only when a search has come to a success (kills the targeted mutant). This approach might be less effective than executing all mutants against all produced tests (fitness evaluations) but keeps mutation execution overheads at a low level. Additionally, for further savings it determines the strongly killed mutants by executing only those that have been previously weakly killed, as pointed out in the introduction section.

### Search based testing

Search based testing (Harman and McMinn 2007), (Wegener et al. 2001) formulates the test case generation problem to a search problem and tries to tackle it using search based optimization techniques. The search process is guided by an appropriate fitness function which indicates how close the tests are in covering the aimed program elements. To achieve this, a separate search is performed according to each live mutant. To keep mutation execution overheads at a low level the framework executes all the live mutants only when a search has come to a success (kills the targeted mutant), as suggested in Fraser and Zeller (2010).

The proposed framework uses the alternating variable method, proposed by Korel (1990). This method forms a hill climbing algorithm which has been shown to be quite effective compared with other search algorithms in the context of structural testing (Harman and McMinn 2007). Hence, it forms an ideal choice as it is a quite simple to implement method and as expected, a quite powerful one. Here, it should be noted that mutation, in particular weak mutation, can be transformed to branch testing (Papadakis and Malevris 2011), (Papadakis et al. 2010) and since hill climbing performs similarly to its rivals, in the structural testing context (Harman and McMinn 2007), there is no reason why this should not hold for mutants too. Nevertheless, this is beyond the scope of the present paper and is left open for future research.

The method starts by randomly initializing the input program variable values. Then it selects repeatedly and adjusts one of those values by alternating it. This is performed until no further fitness improvement can be obtained i.e. no further alternations are fruitful. In this case the method switches to the next input variable. The algorithm stops when no further fitness improvement can be recorded by selecting and alternating any of the input variables. Details of the method can be found in Korel (1990).

### Fitness function

Search based testing requires the employment of an appropriate fitness function in order to be effective. The present framework utilizes a fitness function composed of four parts. The first two are known as the approach level and the branch distance introduced by Wegener et al. (2001) in the context of structural testing. The third one is named mutation distance and the fourth one is named impact distance.

The approach level measures the closeness, of a candidate test case, for executing a target mutant statement. It is calculated by counting the number of the target mutant’s control dependent nodes missed by the candidate input test. The branch distance quantifies the distance from flipping a branch i.e. making it from true to false or the opposite. It is computed using the runtime values of the branch expression of interest. The expression of interest is the topmost of the missed ones from the mutant control dependencies. This measure is calculated based on the expression formulas of Table 1 which was taken from the Awedikian et al. (2009) study on MC/DC testing. Mutation distance as introduced in this paper reflects the branch distance measure on mutants. This approach is in line with the suggestions made by Bottaci (2001) for the mutation testing fitness calculations. It should be noted that these three measures guide the search towards fulfilling the reachability and mutant necessity constraints proposed by Demillo and Offutt (1991). Table 2 presents the expression formulas based on which mutation distance fitness calculations were made. These formulas were obtained by simplifying and reducing the necessity constraints and provide useful information for killing the considered mutants (based on the expression 1). In Table 2 the Ffit(x) and Tfit(x) signify the True and False branch fitness of clause x respectively. Fulfilling the necessity constraints has been found to be relatively ineffective at killing mutants that involve changes to predicate expressions (DeMillo and Offutt 1991). Thus, mutation fitness calculations should quantify the distance of making changes to the mutant and original program predicates (at the mutated statement). To achieve this it is needed to quantify the distance of fulfilling the following expression:

**Table 1:** **Branch fitness**

Expression	True branch	False branch
a == b	abs( a - b)	a == b?k : 0
a ! = b	a ! = b? 0 : k	abs (a ! = b?a - b : 0)
a < b	abs (a < b?0 : a - b + k)	abs (a < b?a - b + k : 0)
a < = b	abs (a < = b?0 : a - b)	abs (a < = b?a - b : 0)
a > b	abs (a > b?0 : a - b + k)	abs (a > b?a - b + k : 0)
a > = b	abs (a > = b?0 : a - b)	abs (a > = b?a - b : 0)
a || b	min[fit(a), fit(b)]	fit(a) + fit(b)
a && b	fit(a) + fit(b)	min[fit(a), fit(b)]

**Table 2:** **Mutation fitness**

Operator	Original expression	Mutant fitness		
*Relational*	a > b	a > = b:***abs(a-b)***	a ! = b:***abs(a-b + k)***	true:***abs(a-b)***
		a < b:***k***	a == b:***abs(a-b)***	false:***abs(a-b + k)***
		a < = b:***0***		
	a > = b	a > b:***abs(a-b)***	a ! = b:***abs(a-b)***	true:***abs(a-b + k)***
		a < b:***0***	a == b:***abs(a-b + k)***	alse:abs(a-b)
		a < = b:***k***		
	a < b	a > b:***k***	a ! = b:***abs(a-b + k)***	true:***abs(a-b)***
		a > = b:***0***	a == b:***abs(a-b)***	false:***abs(a-b + k)***
		a < = b:***abs(a-b)***		
	a < = b	a > b:***0***	a ! = b:***abs(a-b)***	true:***abs(a-b + k)***
		a > = b:***k***	a == b:***abs(a-b + k)***	false:***abs(a-b)***
		a < b:***abs(a-b)***		
	a ! = b	a > b:***abs(a-b + k)***	a < = b:***abs(a-b)***	true:***abs(a-b)***
		a > = b:***abs(a-b)***	a == b:***0***	false:***k***
		a < b:***abs(a-b + k)***		
	a == b	a > b:***abs(a - b)***	a < = b:***abs(a-b + k)***	true:***k***
		a > = b:***abs(a-b + k)***	a ! = b:***0***	false:***abs(a-b)***
		a < b:***abs(a - b)***		
*Arithmetic*	a + b	a - b:***k***	a / b:***k***	a:***k***
		a * b:***k***	a% b:***k***	b:***k***
	a – b	a + b:***k***	a / b:***k***	a:***k***
		a * b:***k***	a% b:***k***	b:***k***
	a * b	a + b:***k***	a / b:***k***	a:***k***
		a - b:***k***	a% b:***k***	b:***k***
	a / b	a + b:***k***	a * b:***k***	a:***k***
		a – b:***k***	a% b:***k***	b:***k***
	a% b	a + b:***k***	a * b:***k***	a:***k***
		a – b:***k***	a / b:***k***	b:***k***
*Absolute*	a	abs(a):***abs(a + k)***	-abs (a):***abs(a)***	0:***abs(a)***
*Logical*	a && b	a||b:***min[Tfit(a) + Ffit(b), Ffit(a) + Tfit(b)]***	a:***Tfit(a) + Ffit(b)***	true:***min [Ffit(a), Ffit(b)]***
			b:***Ffit(a) + Tfit(b)***	false:***Tfit(a) + Tfit(b)***
	a || b	a&&b:***min[Tfit(a)+***	a:***Ffit(a) + Tfit(b)***	true:***Ffit(a) + Ffit(b)***
		***Ffit(b), Ffit(a) + Tfit(b)]***	b:***Tfit(a) + Ffit(b)***	false:***min[Tfit(a), Tfit(b)]***

Following the branch fitness calculations of Table 2, the fitness of the above expression named predicate mutation distance (pmd), is defined according to expression 2.2

The O and M denote the original and the mutant predicates fitness calculations.

Impact distance tries to approximate the mutant sufficiency condition (DeMillo and Offutt 1991), which is a difficult task to formalize (DeMillo and Offutt 1991). Following the suggestions made in Fraser and Zeller (2010), one way of approximating this condition is to measure the impact on the mutant program execution. As this approach does not guide the search towards some specific program parts, able to expose the introduced mutants, it was found to be ineffective with the AVM method. Thus, the proposed fitness function tries to guide the search towards some specific program elements which will hopefully be capable of exposing the mutants. To achieve this, it is suggested to record the impact (differences on the execution paths between the original and mutant program versions) (Fraser and Zeller 2010) of each mutant during the test generation process. For each program node that has been impacted the ratio of the killed over the total number of mutants is recorded. Informally, as tests are produced and executed with mutants the nodes are ranked according to their ability to expose mutants, when they are impacted. Impact distance reflects the approach level and the branch distance on the mutant program towards covering a selected top ranked node.

Conclusively, the proposed fitness function guides the search towards reaching (approach level + branch distance) a mutant, causing a discrepancy at the mutation point (mutation distance), propagate it to the outcome of the mutant statement (predicate mutation distance) and impact specific likely to expose mutants, program nodes (here referred to as impact nodes). Computing the overall fitness of the test cases requires a unification of the three used measures. This is done based on the following equation where branch and mutation distances are normalized as in (Arcuri 2010):3

### Dynamic approach level

Mutation testing introduces a vast number of mutants which are spanned across the whole program structure. It was observed that trying to kill them, results into covering - reaching many other mutants (possible hard to reach) collaterally i.e. without aiming at them. It is noted that many mutants are equivalent and thus by their definition aiming at them will result in a waste of effort. In practice, these two characteristics of mutation can provide useful information to assist the killing of some other mutants. This paper proposes the concept of dynamic approach level that will serve as a yardstick for improving the search process.

Search based approaches utilizing approach level and branch distance fitness functions have the drawback of leading to random testing (McMinn and Holcombe 2006) for certain types of programs. This is due to the use of certain programming constructs such as the use of flags, enumeration types and various data dependencies (Awedikian et al. 2009). To avoid such a situation, the dynamic approach level tries to include some data dependencies in the fitness evaluation. These data dependencies are not included in the “static” predefined approach level. The need for such an inclusion has also been pointed out by Awedikian et al. (2009), who also argued that doing so is quite easy and leads to an improved performance.

The rationale behind the use of the standard approach level (Wegener et al. 2001) is to include only the structural elements (control dependencies) that must be traversed by any of the possible sought test cases. Consider a case where in order to traverse a targeted branch requires the program execution to execute a specific program statement (data dependency) that is not part of the control dependencies of the targeted branch. Then, all the possible test cases that traverse this branch also traverse the specific program statement. The dynamic approach level identifies all the common structural elements that traverse the produced test cases and thus, necessary data dependencies too. This way the path information gained during the whole search process can be used and reproduced for infecting and eventually killing the aimed mutants.

The dynamic approach level is defined as the intersection of all nodes that are contained in all the encountered execution paths that reach a targeted node. Thus, for example if a target node is x and during the search process 5 different execution paths have been encountered that lead to node x, then the dynamic approach level is formed as the common nodes of these 5 paths. If there is no path leading to node x, then the standard approach level is used. It is noted that in the absence of data dependencies the dynamic approach level could approximate the standard approach level if most of the encountered paths have been executed. This approach relies on the excessive search performed to kill all the introduced mutants.

### The mutation AVM method

The proposed approach uses the alternating variable method, proposed by Korel (1990). This method forms a Hill climbing algorithm which has been shown to be quite effective compared to other search algorithms in the context of structural testing (Harman and McMinn 2007) and has also been incorporated to automated test data generation tools such as the AUSTIN tool (Lakhotia et al. 2010) for structurally testing. Hence, it forms an ideal choice as it is a quite simple to implement method and as it is also expected to be a quite powerful one. Here, it should be noted that mutation testing, in particular weak mutation, can be transformed to branch testing (Papadakis and Malevris 2009), (Papadakis et al. 2010), (Papadakis and Malevris 2011), (Papadakis and Malevris 2012) and since hill climbing performs similarly to its rivals, in the structural testing context (Harman and McMinn 2007), there is no reason why this should not hold for mutants too. Nevertheless, this is beyond the scope of the present paper and is left open for future research.

**Figure 1:**
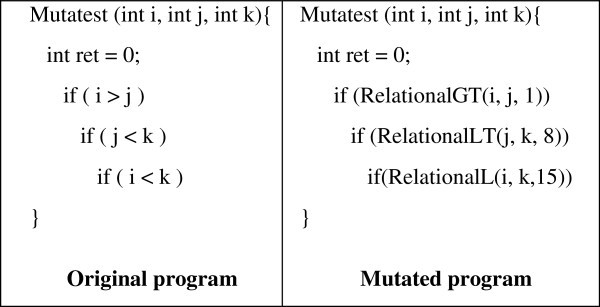
**Demonstrating Example.**

The method starts by randomly initializing the input program variable values. Then it selects repeatedly and adjusts one of those values by alternating it. This is performed until no further fitness improvement can be obtained i.e. no further alternations are fruitful. In this case the method switches to the next input variable. The algorithm stops when no further fitness improvement can be recorded by selecting and alternating any of the input variables. Consider the example of Figure 1. To make this example more understandable, let us assume that when a mutant is weakly killed, it is also strongly killed. The same approach holds and in the opposite case with the difference in the fitness calculations. In the left part of Figure 1 the original sample program is presented. In its right part the mutated meta-program is detailed. The introduction of the mutants is recorded in the alterations made to the original program e.g. the statement if ( i < k ) has become if (RelationalGT(i, k, 15)). The variables i and j are the two original operand variables while 15 signifies that this expression contains the mutants identified by the relational operator (7 mutants) with identification numbers from 15 to 21. Let the initial random inputs be: i = 150, j = 400, k = 300 and the target mutant the 15th one i.e. ( i < k to i < = k with mutant fitness abs(i–k)). The process at first selects the i input variable and performs exploratory steps (small increases and decreases say p - here 1 for integer and 0.1 for real variables - of the input variable). These steps indicate the search direction. In the example here, i should be increased as it results in better fitness values. After the determination of the search direction the process continues with pattern steps (these steps are computed based on the formula: 2^n*direction*p, where direction is 1 for increase or −1 for decrease). Thus, in the above example the next obtained input values (pattern steps) will be for the i variable 152, 154, 158, 166, 182, 214, 278, 406. At this point the fitness function cannot be further improved by altering the i input variable as the fitness also relies on the second branch point ( j < k ). The process continues with input variable j, it performs exploratory steps and starts to decrease the j value as follows: 398, 396, 392, 384, 368, 336, 272. At that point the process chooses the k input variable and starts increasing its value accordingly to 302, 304, 308, 316, 332, 364, 428. After value 428 it performs exploratory steps again and starts to decrease its value to 426, 424, 420, 412, 396. Here it changes direction again and continues to 398, 400, 404, 412 where it decreases to 410, 408, 404 and finally finds the required value 406 that kills the mutant. The process has effectively achieved to produce the test case ( i = 406, j = 272, k = 406 ) that kills the mutant ( i < k to i < = k ). If this procedure fails to kill the required mutant the process restarts by using new randomly selected inputs for i, j and k. Of course, this could be a consequence of hitting a local minimum or a consequence of an equivalent mutant.

## Evaluation

This section empirically evaluates the effectiveness of the propositions made in this paper based on two experiments. The first experiment compares the effectiveness of the proposed framework to perform mutation using three fitness functions and random testing. The second experiment, examines the impact on the framework’s effectiveness to when utilizing the dynamic approach level.

### Experimental design

The experiment described in this section uses the proposed mutation testing framework on a set of Java programs using the mutation operators presented in Table 2 along with the incorporated fitness necessity formulas. The framework works on Java programs (produces mutation operators) with a primary target at the intra method level (Ma et al. 2005), similar to non Object-Oriented languages (see section V.A for details about the framework capabilities and limitations). The experiment described in this section empirically investigates the following Research Questions (RQs):

-  RQ 1: How effective are the adopted fitness functions compared to a previously proposed one (Ayari et al. 2007) and random testing?
-  RQ 2: What is the relative efficiency of the adopted fitness functions?
-  RQ 3: What is the impact of the dynamic approach level on the effectiveness of the examined approaches?

To answer the above questions, the proposed framework was employed to generate test cases for a set of programs based on the mutation operators presented in Table 2. It is noted that the results reported here are the average values obtained from applying the examined approaches 10 times independently. In order to answer RQ1 the number of killed mutants was measured. With respect to RQ2 the required fitness evaluations to produce the sought test data were measured. With respect to RQ3 the experiment was repeated by utilizing the dynamic approach level. Specifically, in the conducted experiments random testing and three fitness functions were utilized. The first fitness function named “Reach” uses only the reach distance of expression 3 and corresponds to the fitness function suggested by Ayari et al. (2007). The second one called “Infect” uses the Reach and Infect distances of expression 3 and the third one named “Impact” utilizes expression 3. In the second experiment the followed process was to iteratively and continuously perform one attempt to kill all the live mutants using the standard approach level and one using the dynamic approach level. For each test subject the test generation process considered up to 50,000 fitness evaluations or random test generations (for random testing) for all the introduced mutants. This is a considerably high number of evaluations but it is forced due to the existence of equivalent mutants.

### Results and analysis

The experimental evaluation of the proposed system was performed based on the use of 8 program units. The selected programs have been used in various studies (DeMillo and Offutte^1991^), (Papadakis et al. 2010), (Sthamer 1996), (Murrill 2008). The test objects are programs with various characteristics such as mathematical computations, array manipulations, state based behavior and complex branching conditions. A considerable number of mutants, 2,759, were produced based on the use of all the operators employed by the framework. Particulars of all the used programs are given in Table 3. Table 3 records details about the test objects’ lines of code, input settings (input domain search space) and the number of produced mutants.

**Table 3:** **Test program details**

Test subjects	Lines of code	Input settings	No. of mutants
*1-Triangle*	*35*	*3 ints: (range 16-bit)*	*166*
*2-Trityp*	*40*	*3 ints: (range 16-bit)*	*349*
*3-Triangle*	*90*	*3 ints: (range 16-bit)*	*421*
*4-Remainder*	*50*	*2 ints: (range 16-bit)*	*324*
*5-Callendar*	*75*	*5 ints: 2x[0, 12], 2x[0, 365], [−3,000, 3,000]*	*327*
*6-Cancel*	*50*	*3 ints: (3x[0, 50])*	*866*
*7-FourBalls*	*30*	*4 floats, 1 ints: 4x[−100, 100], [−100, 100]*	*225*
*8-Quadratic*	*25*	*3 ints (range 16-bit)*	*81*

The experiment tries to reveal the ability of the framework to perform mutation testing and its effectiveness compared to a previously proposed approach without any particular assistance. That is, none of the equivalent mutants was eliminated from the candidate mutant set, fact that allows considerable overheads to the conducted experiment. Furthermore, no data dependencies, state related information or flag removal approaches were employed in order to make the test search more efficient. The effective incorporation of such approaches is considered out of the scope of the present paper and thus, has been left for future work.

**Figure 2:**
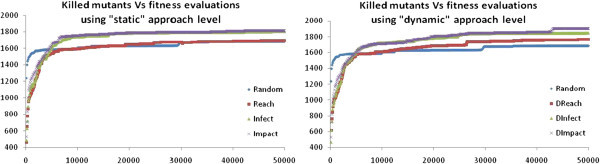
**Mutants killed by the utilized approaches for various fitness evaluation limits.**

Results of the performed experiments are presented in Table 4. Table 4 records per test subject the number of killed mutants by the produced test data according to random testing (Random) and the three employed fitness functions utilizing the standard (Reach, Infect and Impact) and the dynamic approach levels (DReach, DInfect and DImpact). Additionally, Figure 2 reports the sum of the killed mutants for various fitness evaluation limits when using either static or dynamic approach level.

**Table 4:** **Mutants killed by the utilized fitness functions**

Test subjects	Random	Reach	Infect	Impact	DReach	DInfect	DImpact
***1-Triangle***	***102.2***	***94***	***103***	***103.4***	***96.4***	***103***	***103.2***
***2-Trityp***	***125.6***	***173.8***	***178.4***	***184.8***	***205.4***	***210.4***	***223***
***3-Triangle***	***102***	***131***	***144.4***	***146.2***	***143.8***	***148.6***	***185***
***4-Remainder***	***205.8***	***201.4***	***206***	***206***	***201.4***	***206***	***206***
***5-Callendar***	***189***	***165***	***195.2***	***193.2***	***168.6***	***198.8***	***200***
***6-Cancel***	***712.6***	***686.2***	***732.2***	***732.6***	***709.26***	***732***	***733.2***
***7-FourBalls***	***187.2***	***183.2***	***185***	***186.8***	***181***	***185.8***	***188***
***8-Quadratic***	***59.07***	***58***	***61.22***	***61.8***	***58***	***60.6***	***63***

The obtained results provide evidence in support of the proposed fitness functions (Infect and Impact) which outperform a previously proposed one (Reach) and random testing (RQ1). Additionally, the use of dynamic approach level improves the effectiveness of all the examined fitness functions (RQ3). Specifically, the Infect and Impact fitness functions kill on average 113 and 122 more mutants than the Reach one respectively, using the standard approach level. The use of the dynamic approach level results in an increase with all three examined functions by killing 71, 40 and 87 more mutants for Reach, Infect and Impact fitness functions respectively. Additionally, the convergence of all the examined fitness functions is higher for the high number of evaluations. This is due to the fact that for higher number of executions more paths are included in the dynamic approach level. Recall that the dynamic approach level is adopted according to all the encountered execution paths.

Considering the approach efficiency (RQ2) the number of mutant evaluations should be examined. From Figure 2 it can be observed that both the Infect and Impact fitness are more efficient than the Reach one even for a small number of evaluations (approximately over 4,500 evaluations). For less than 4,500 evaluations all the approaches share a similar effectiveness and efficiency. The use of dynamic approach level generally improves the efficiency of the utilized fitness functions as for the same number of evaluations it kills more mutants.

Compared with random testing it can be observed that in general it performs worse than the Infect and Impact fitness irrespective of the use or not of the dynamic approach level. However, it performs similarly to Reach without the use of the dynamic approach level and worsens when Reach utilizes it. Here it must be noted that under the framework’s process of executing mutants, which determines the collaterally killed ones when and only when it has killed a targeted mutant, the comparison made is in favor of random testing (in random testing all tests are executed against all mutants). Nevertheless, even in such a case the proposed approach outperforms random testing.

**Figure 3:**
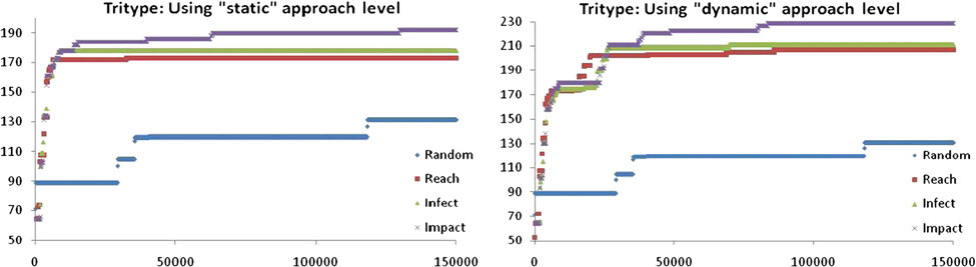
**Trityp program: Mutants killed vs fitness evaluations.**

By employing the proposed framework with 150,000 fitness evaluations on the Trityp program (DeMillo and Offutt 1991), which is a well established benchmark in both search based and mutation testing studies, the results presented in Figure 3 can be obtained. From this figure it becomes evident that the Impact fitness utilizing the dynamic approach level can lead to a considerably high number of killed mutants. In this case it manages to kill all but two of the killable mutants. This fact suggests that the proposed approach can be quite powerful in producing mutation based test cases.

## Related work

The alternating variable method was initially proposed by Korel (1990) which was adopted by the present framework for finding the appropriate tests. Daimler (Wegener et al. 2001) developed an automated tool for testing C programs based on various structural testing criteria. It is this tool’s fitness function that is extended by the present research.

Test case generation techniques for mutation testing have received little attention in the literature. The most fundamental attempt is the one due to DeMillo and Offutt who developed the constraint based testing Technique (DeMillo and Offutt 1991). This technique introduced the concept of reachability, necessity and sufficiency conditions which have been embodied in a tool called Godzilla. Godzilla contains the first two of these conditions, formulating and resolving them as mathematical systems of constraints. Formulating and resolving reachability and necessity constraints forms a difficult task. In order to efficiently handle this task, in (Papadakis and Malevris 2012) and (Papadakis and Malevris 2009) it is suggested to use a path selection strategy that reduces the effects of infeasible paths. Bottaci (2001) proposed a fitness function composed of the reachability distance (measures the closeness of the test data and the mutant statement) of the produced tests and the necessity distance (measures the closeness to kill the mutant statement). In (Ayari et al. 2007) a search based approach for the generation of mutation test data was proposed by implementing only the reachability part of the Bottaci (2001) fitness function. More recently, Fraser and Zeller (2010) proposed another evolutionary based approach to automate the production of mutation tests. This approach uses the rechability part of the Bottaci’s fitness function (Bottaci 2001) and approximates the necessity and sufficiency conditions by measuring the mutant’s impact (Fraser and Zeller 2010). They argue that producing tests with higher mutants’ impact, results in tests closer to kill those mutants. The above two approaches are the closest ones to the present proposed framework. The main differences are that the proposed framework extends the fitness function to effectively direct the search towards necessity and sufficiency conditions (DeMillo and Offutt 1991). Additionally, a novel technique to efficiently gain and dynamically adopt the required fitness information through mutant schemata is presented by the present paper.

The idea of utilizing mutant schemata in order to help automated tools to perform mutation was initially introduced in (Papadakis et al. 2010) with the aim of reusing existing structural automated tools for performing mutation. The underlying technique to achieve this was to reduce the weakly killing mutant problem to the covering branches one. In (Papadakis and Malevris 2010a) the schemata approach was extended to utilize dynamic symbolic execution for producing strong mutation based tests. In the present paper, mutant schemata were used in order to enable the fitness guidance towards killing mutants for strong mutation.

## Discussion

The origin of the proposed framework is due to the integrated use of mutant schemata and evolutionary testing techniques utilizing a novel fitness function. This integration helps to extract dynamic program information concerning the introduced mutants and fitness calculations efficiently. The conducted case study indicates the ability of the proposed method to produce high quality test cases from scratch (starting from random inputs). Additionally, it indicates the improved performance over random testing and a previously proposed approach. In fact the proposed fitness functions are compared with the one proposed in (Ayari et al. 2007). A similar to (Ayari et al. 2007) approach has also been proposed by Fraser and Zeller (2010) who extend it by including the mutants impact in their fitness. Such an inclusion was attempted in the conducted case study. The obtained results were similar to the ones obtained by the reach fitness function. This is due to the use of hill climbing and the absence of effective guidance towards some specific program statements.

Equivalent mutants help the proposed approach to build the dynamic approach level as they force the search towards various and different program statements and conditions. Despite this, from the conducted case study it becomes evident that equivalent mutants pose an additional burden to test case evolution as they force the method not only to search for non killable mutants (huge effort) but also by misleading the mutation score calculated due to their presence. This fact explains why the proposed approach spends so many fitness evaluations in order to kill additional mutants. Perhaps the use of some heuristic approaches such as the one suggested in (Kintis et al. 2012) for isolating equivalent mutants, could be employed in order to overcome this problem. This paper also reveals that simple dynamic approaches can be quite effective for the production of high quality test cases. Based on the dynamic nature of the adopted approach, the problems caused by pointers and non linear expressions are limited.

The framework described here uses a quite simple but practical approach, based on the mutant schemata technique (Untch et al. 1993), in order to perform the test data generation process. This approach is an incremental approach that targets first on reaching, then weakly killing and then strongly killing the introduced mutants. This incremental process helps on producing some tests capable to weakly kill some strongly equivalent mutants. These tests should be valuable and should increase the performed testing quality.

### Tool characteristics and limitations

The proposed framework in this paper has several special characteristics and limitations which are currently under research. Generally, it can handle Java programs only at the intra method level. Thus, it does not handle method sequences or Object Oriented features. Its application treats one method at a time using predefined method sequences. The inability of the mutant schemata technique to handle certain Object Oriented mutants as identified in (Ma et al. 2005) limits the propositions made in this paper to the intra method level. Here it must be noted that in the case of Logical operators, a necessary special handling was enforced. This is due to the short circuit evaluation mechanism performed by the Java language. In order to keep the program execution paths unaffected with the presence of mutants, the logical operator’s evaluations were performed when both logical operands were executed.

### Threats to validity

The present paper focuses on presenting an automated mutation testing system. One possible threat to the validity of the results reported here may be related to the generalization of the obtained results. Thus, the framework’s effectiveness may vary in other cases. However, as the proposed method utilizes and extends the suggestions made by DeMillo and Offutt (1991), Bottaci (2001) and Fraser and Zeller (2010), their use is expected to increase the effectiveness of the search based approaches. Additionally, the results obtained may serve as a yardstick towards the employment of mutation testing in an automated fashion and in order to indicate that it is possible to adopt mutation for the testing activity, resulting in the production of high quality tests.

The proposed framework uses weak/strong mutation and mutants’ impact for guiding and evaluating its test production effectiveness utilizing the AVM method. This does not necessarily mean that the results here can be extrapolated to the rest of the search based approaches. In any case, this was not the intention of the present framework, as it aims at using AVM which is quite simple and practical.

The last threat of internal validity may be based on the use of software systems. Thus, possible bugs, the fitness measures and the schematic program production implementation of the test objectives may have influenced the obtained results. To reduce these threats, selected test cases were executed in both the original and the converted program versions showing that they execute the same program paths. An additional manual evaluation of the results produced by the framework based on the Tritype program was performed showing no discrepancies.

## Conclusion and future work

The proposed framework, as described here forms an automation of the mutation testing method. The framework uses state of the art techniques to efficiently generate the candidate mutants and produce mutation based test data. Based on a performed case study the system achieves to produce test cases able to kill the majority of the introduced mutants. This also establishes tests for performing high quality testing, this being the main issue of the present paper. Preliminary results suggest that the proposed fitness functions can outperform a previously proposed one and random testing as well. Also the use of the dynamic approach level can increase the effectiveness of the framework. In particular, based on the conducted case study, the suggested propositions achieve to kill on average approximately 7.6% more mutants than a previously proposed approach (Ayari et al. 2007) and 7.9% more than what random testing does.

In future, extensions of the framework to include other search based approaches such as evolutionary testing are planned. Further investigation is needed in order to determine the benefits of the dynamically adopted approach level and its optimal use on search based testing. Finally, the application of the proposed approach in killing second or higher order mutants (Papadakis and Malevris 2010b), (Kintis et al. 2010), (Jia and Harman 2010) is under investigation. Since such approaches have been shown to be quite effective in isolating equivalent mutants (Kintis et al. 2012) their consideration within the proposed framework will greatly enhance the level of automation used when performing mutation testing.

## References

[CR1_205] Andrews JH, Briand LC, Labiche Y, Namin AS (2006). Using mutation analysis for assessing and comparing testing coverage criteria. IEEE Trans Softw Eng.

[CR2_205] Arcuri A (2010). It Does Matter How You Normalise the Branch Distance in Search Based Software Testing. Proceedings of the 2010 Third International Conference on Software Testing, Verification and Validation.

[CR3_205] Awedikian Z, Ayari K, Antoniol G (2009). Mc/dc automatic test input data generation.

[CR4_205] Ayari K, Bouktif S, Antoniol G (2007). Automatic mutation test input data generation via ant colony. Proceedings of the 9th annual conference on Genetic and evolutionary computation.

[CR5_205] Bottaci L (2001). A genetic algorithm fitness function for mutation testing. SEMINAL: Software Engineering using Metaheuristic INovative Algorithms, Workshop 8, ICSE 2001.

[CR6_205] DeMillo RA, Offutt AJ (1991). Constraint-based automatic test data generation. IEEE Trans Softw Eng.

[CR7_205] DeMillo RA, Lipton RJ, Sayward FG (1978). Hints on test data selection: Help for the practicing programmer. Computer.

[CR8_205] Fraser G, Zeller A (2010). Mutation-driven generation of unit tests and oracles. Proceedings of the 19th international symposium on Software testing and analysis, Trento, Italy.

[CR9_205] Hamlet RG (1977). Testing programs with the aid of a compiler. IEEE Trans Softw Eng.

[CR10_205] Harman M, McMinn P (2007). A theoretical & empirical analysis of evolutionary testing and hill climbing for structural test data generation. Proceedings of the 2007 international symposium on Software testing and analysis, London, United Kingdom.

[CR11_205] Howden WE (1982). Weak mutation testing and completeness of test sets. IEEE Trans Softw Eng.

[CR12_205] Jia Y, Harman M (2010). An analysis and survey of the development of mutation testing. IEEE Trans Softw Eng.

[CR13_205] Kintis M, Papadakis M, Malevris N: *Evaluating Mutation Testing Alternatives: A Collateral Experiment. Proceedings of the 2010 Asia Pacific Software Engineering Conference*. Washington, DC, USA: IEEE Computer Society; 2010:300–309. [*APSEC '10*] doi:10.1109/APSEC.2010.42 mutation testing, higher order mutation, weak mutation, collateral coverage. ISBN 978–0-7695–4266–9

[CR14_205] Kintis M, Papadakis M, Malevris N: *Isolating First Order Equivalent Mutants via Second Order Mutation. Proceedings of the 2012 IEEE Fifth International Conference on Software Testing, Verification and Validation*. Washington, DC, USA: IEEE Computer Society; 2012:701–710. doi:10.1109/ICST.2012.160 Equivalent mutants, Higher Order mutation, Mutants' Impact. ISBN 978-0-7695-4670-4

[CR15_205] Korel B (1990). Automated software test data generation. IEEE Trans Softw Eng.

[CR16_205] Lakhotia K, Harman M, Gross, Austin H (2010). A tool for search based software testing for the c language and its evaluation on deployed automotive systems. 2nd International Symposium on Search Based Software Engineering.

[CR17_205] Ma Y-S, Offutt J, Kwon YR (2005). Mujava: An automated class mutation system. Softw Test Verif Reliab.

[CR18_205] McMinn P, Holcombe M (2006). Evolutionary testing using an extended chaining approach. Evol Comput.

[CR19_205] Murrill BW (2008). An empirical, path-oriented approach to software analysis and testing. J Syst Softw.

[CR20_205] Offutt AJ, Pan J (1997). Automatically detecting equivalent mutants and infeasible paths. Software Testing, Verification and Reliability.

[CR21_205] Offutt AJ, Untch RH, Eric WW (2001). Mutation 2000: uniting the orthogonal. Mutation testing for the new century.

[CR22_205] Papadakis M, Le Traon Y: *Using Mutants to Locate “Unknown” Faults. Proceedings of the 2012 IEEE Fifth International Conference on Software Testing, Verification and Validation*. Washington, DC, USA: IEEE Computer Society; 2012:691–700. [*ICST '12*] doi:10.1109/ICST.2012.159 debugging, mutation analysis, fault localization

[CR23_205] Papadakis M, Malevris N (2009). An effective path selection strategy for mutation testing. Proceedings of the 16th Asia-Pacific Software Engineering Conference.

[CR24_205] Papadakis M, Malevris N: *Automatic Mutation Test Case Generation via Dynamic Symbolic Execution. Proceedings of the 2010 IEEE 21st International Symposium on Software Reliability Engineering*. Washington, DC, USA: IEEE Computer Society; 2010:121–130. [*ISSRE '10*] http://dx.doi.org/10.1109/ISSRE.2010.38 10, 1914368, automated test case generation, dynamic symbolic execution, mutation testing, mutant schemata 10.1109/ISSRE.2010.38

[CR25_205] Papadakis M, Malevris N (2010b). An empirical evaluation of the first and second order mutation testing strategies. Software Testing, Verification, and Validation Workshops (ICSTW), 2010 Third International Conference on, 6–10 April 2010.

[CR26_205] Papadakis M, Malevris N (2011). Automatically performing weak mutation with the aid of symbolic execution, concolic testing and search-based testing. Softw Qual J.

[CR27_205] Papadakis M, Malevris N (2012). Mutation based test case generation via a path selection strategy. Inf Softw Technol.

[CR28_205] Papadakis M, Malevris N, Kallia M (2010). Towards automating the generation of mutation tests. Proceedings of the 5th Workshop on Automation of Software Test, Cape Town, South Africa.

[CR29_205] Sthamer HH (1996). The automatic generation of software test data using genetic algorithms.

[CR30_205] Untch RH, Offutt AJ, Harrold MJ (1993). Mutation analysis using mutant schemata. Proceedings of the 1993 ACM SIGSOFT international symposium on Software testing and analysis, Cambridge, Massachusetts, United States.

[CR31_205] Wegener J, Baresel A, Sthamer H (2001). Evolutionary test environment for automatic structural testing. Inf Softw Technol.

